# Computation of Pacemakers Immunity to 50 Hz Electric Field: Induced Voltages 10 Times Greater in Unipolar Than in Bipolar Detection Mode

**DOI:** 10.3390/bioengineering4010019

**Published:** 2017-03-06

**Authors:** Cihan Gercek, Djilali Kourtiche, Mustapha Nadi, Isabelle Magne, Pierre Schmitt, Martine Souques

**Affiliations:** 1 Institut Jean Lamour, Université de Lorraine-CNRS (UMR 7198), BP 70239, 54506 Vandœuvre, France; djilali.kourtiche@univ-lorraine.fr (D.K.); mustapha.nadi@univ-lorraine.fr (M.N.); pierre.schmitt@univ-lorraine.fr (P.S.); 2 EDF R&D, Avenue des Renardières–Ecuelles, 77818 Moret-sur-Loing, France; isabelle.magne@edf.fr; 3 EDF SEM, Immeuble Carré Vert, 45 rue Kleber, 92309 Levallois-Perret CEDEX, France; martine.souques@edf.fr

**Keywords:** electric field, low frequency, active implantable medical devices, implanted cardiac defibrillators, pacemaker, cardiac implants

## Abstract

Thisstudy aims to compute 50 Hz electric field interferences on pacemakers for diverse lead configurations and implantation positions. Induced phenomena in a surface-based virtual human model (standing male grounded with arms closed, 2 mm resolution) are computed for vertical exposure using CST EM^®^ 3D software, with and without an implanted pacemaker. Induced interference voltages occurring on the pacemaker during exposure are computed and the results are discussed. The bipolar mode covers 99% of the implanted pacing leads in the USA and Europe, according to statistics. The tip-to-ring distance of a lead may influence up to 46% of the induced voltage. In bipolar sensing mode, right ventricle implantation has a 41% higher induced voltage than right atrium implantation. The induced voltage is in average 10 times greater in unipolar mode than in bipolar mode, when implanted in the right atrium or right ventricle. The electric field threshold of interference for a bipolar sensing mode in the worst case setting is 7.24 kV·m^−1^, and 10 times higher for nominal settings. These calculations will be completed by an in vitro study.

## 1. Introduction

Recent publications of the USA and European authorities point out that cardiovascular diseases are the principal cause of death, in agreement with the worldwide WHO report of 2014 [1,2,3]. When cardiovascular disease is caused by cardio-electrical system dysfunction, one of the possible clinical treatments is an active cardiac device implantation, as pacemakers which treat especially slow-rated arrhythmias. Constituted of a metallic box with electronic circuits and a battery inside, the pacemaker performs the stimulation/detection in the heart, via its lead. It has two different modes of detection: the unipolar mode which uses the metallic box as an electrode (anode) and the lead as another electrode (cathode), contrary to the bipolar mode, which employs the two electrodes of the lead. In the USA, 2.9 million pacemakers were implanted in patients, with a 55% increase between 1993 and 2009 [4]. An active cardiac implant manufacturer’s technical services department indicates that they receive +60,000 calls per year from physicians or clinicians, and 8% of them are estimated to be about electromagnetic compatibility (EMC) [5]. Moreover, the same company receives +6500 calls/month from patients, 28% of which are related to EMC [6]. These statistics concretely show the increasing number of patients and the concerns of the public, including clinicians and physicians, associated with electromagnetic interferences (EMI).

The European Directive 2013/35/EU defines 50 Hz--induced electric field values in the human body as exposure limit values, to protect from possible adverse health effects caused by external 50 Hz electric and magnetic fields [7]. This directive defines implanted workers as being “at particular risk”. Related European standards (EN-50527-1, EN-50527-2-1) propose several methods, including a numerical approach, to analyze the risks of EMI for pacemakers [8,9]. This article will only deal with the exposure to 50 Hz electric fields, as the magnetic fields and their interactions with implantable cardiac defibrillators, were dealt with in a previous study [10].

The analytical approaches proposed by EN 50527-2-1 to calculate the approximate interference voltages for a frequency range between 16 and 60 Hz electric field, in unipolar and bipolar sensing modes, are Equations (1) and (2):

(1) $$\mathrm{Unipolar}:V_{pp}^{ind,max}=4.4\times 10^{-9}\times E_{p}\times f$$

(2) $$\mathrm{Bipolar}:V_{pp}^{ind,max}=2.3\times 10^{-10}\times E_{p}\times f$$

where $V_{pp}^{ind,max}$ is the maximum induced voltage peak to peak, sensed by the pacemaker measured peak to peak (V); $E_{p}$ is the peak amplitude of the uniform external electric field (V/m); and *f* is the frequency (Hz). The constants (m·s) are deduced from the inductions on the detection curve of the homogeneous human body model, for the frequency range [9]. These equations only provide approximate results, due to being deduced from geometrically simplified homogeneous human models. The authors claim that the approximation shows results which are comparable to sophisticated models [11,12].

Although the Finite Difference Time Domain (FDTD) and Maxwell Grid Equations (MGE) were introduced in 1966 [13], one of the first promising computational methods to define the 3D inductions of electric fields inside a human model, was based on the impedance method, by making a 3D impedance network in 1984 [14]. It took more than ten years for FDTD to be used for estimating the surface charge densities caused by an external 60 Hz electric field [15]. The same method was also used at 10 MHz, with 60 Hz conductivities, to linearly scale the results to 60 Hz [16]. The computational power that the FDTD method needed at that time was huge, so, diverse methods have been proposed. The scalar potential finite-difference method (SPFD) permitted the calculation of these fields more rapidly, by requiring less memory [17]. Hybrid approaches have been used to calculate induced electric fields in a child model, for the exposure to an external electric field [18]. Few studies have focused on the surface-based model, which respect to the increasingly curved geometry of the tissues, when compared to voxel cubes [19]. An extensive bibliographic analysis of the subject, for both an electric and magnetic 50 Hz field, was recently proposed in [20].

Even if the first studies on the compatibility between pacemakers and 50/60 Hz electric fields date from 1964, only 22 scientific papers in English have dealt with the subject, according to the EMF-Portal [21]. Most of them use analytical, in vitro, and in vivo approaches; in fact, these approaches do not analyze the electric field alone. To our knowledge, only two studies have numerically investigated the 3D external electric fields of 50/60 Hz and the EMI on pacemakers. The first study dates back to 2005 and numerically investigated 60 Hz vertical electric fields and the EMI on pacemakers, using a virtual human model [22]. The authors used a 3.6 mm resolution voxel human. The lead cable was ignored and the lead electrodes were represented in voxels, which were consequently larger than real electrodes. For 60 Hz, the range of induced voltages on the pacemaker electrodes (bipolar min.–unipolar max.: 0.056 mV–0.73 mV) were much greater than the analytical solution values (bipolar–unipolar: 0.014 mV–0.26 mV). Another point is the difference between the unipolar and bipolar sensing modes, which was up to a factor of four for this study, when this difference was close to 20 in the analytical formulas (as Equations (1) and (2)). The second study is recent and has numerically analyzed the angle of electrode lead, to proceed with experiences in vivo [23], as was exhibited in a previous study with the current injection [24]. The numerical section mentions an exclusively bipolar configuration with a simplified human model. The influence of the probe position as a percentage, with one single type of lead, has been announced [23].

Our study aims to define the interferences threshold occurring during an exposure of an anatomically implanted human model to a 50 Hz electric field, by using the Finite Integral Technic (FIT) method [25]. The realistic configurations of divers, announced by lead manufacturers, will be compared.

## 2. Methodology

**Figure 1:**
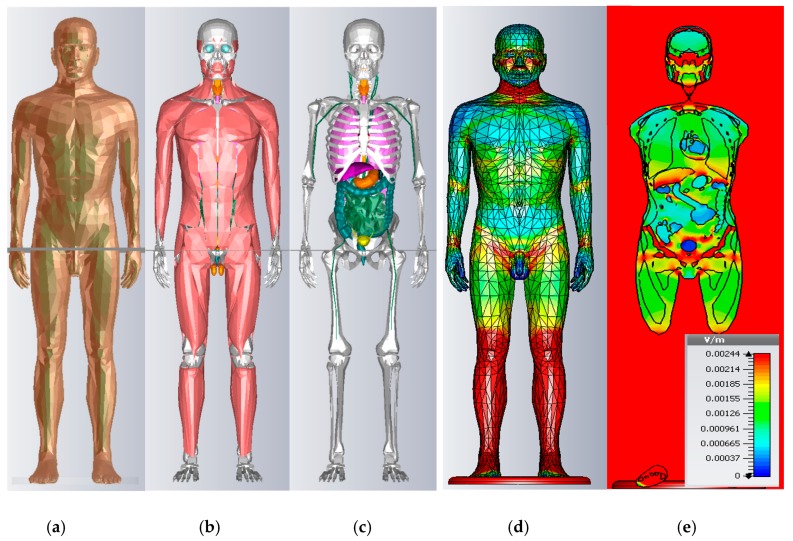
Human body model images at a resolution of 2 mm from left to right, respectively, (**a**) the skin is visible (left), (**b**) the skin and fat layers have been hidden and (**c**) the muscle layer has been hidden to show the internal organs (middle); Electric field induced on the model under 1 kV/m 50 Hz, (**d**) whole body in 3D and (**e**) coronal view (right).

CST EM^®^ software, based on the FIT method, is used with the electroquasistatic domain assumptions and tetrahedral meshes [25,26]. The numerical iteration is set to a precision of 10^−6^ and the total mesh number ranges from 8M up to 28M tetrahedron. We have used a 2mm resolution, as recommended by [27]. In this part, the validation of our calculation method and our human body model are presented. A homogeneous half ellipsoid phantom (σ = 0.2 S/m, grounded), defined by the IEC 62226-3-1 standard, was simulated under a 50 Hz electric field, and the same total current density was obtained with the standard results derived with analytical formulas (J = 0.134 mA·m^−2^, Figure 1a) [28]. The currents induced in the axis-symmetric model were numerically calculated with an error of 0.73% for the current density induction in the neck, to compare to the results given in the IEC 62226-3-1 standard [28]. In this way, we have validated the numerical method according to the analytical solutions and standard, and the virtual human model will be validated in the following sections.

### 2.1. Description of Virtual Human Model: Ansoft

Contrary to surface-based models [19], the construction of the voxelised human body diffracted in equal cubes is less time consuming, but amplifies numerical errors called staircasing effects [20]. Our surface-based model is 183.6 cm tall, presenting a realistic anatomical male model of the human body in a standing position, with the arms closed on the sides and the feet grounded (ANSOFT, ANSYS^®^, Canonsburgh, PA, USA) (Figure 1). The model is almost +1σ taller than the ICRP reference male [29], constructed with 272 different organs and 32 different isotropic conductivity and permittivity tissue values. These properties are similar to the properties of the “Virtual Family” of the IT’IS database, which values are given subsequently [30]. An illustration of the model in 3D is shown in Figure 1, the aspect of inductions can be seen in Figure 1d, and a coronal view is presented in Figure 1 e for the internal organs.

### 2.2. Validation of Numerical Methods with Ansoft Model

The total current travelling to the ground may be estimated analytically, using the following equation [31]:

(3) $$I=5.4\times 10^{-9}\times E_{p}\times h^{2}$$

where *h* is the height of the human body (m), *I* is the measurable total current (A), and $E_{p}$ is the peak amplitude of the uniform external electric field (V/m). The constant (A/(V·m)) is empirically derived from the equivalent charge collecting area of a human (2πf × ε × π × tan^2^(35.7)). The formula considers the human body as homogeneous and spherical. As measurements inside the human body are impossible for ethical reasons, this external value which is also measurable in vivo, has been taken as a value of verification for numerical investigations and for virtual models [19]. By using simulations, the induced total current density, integral over the contact surface between the feet and ground, gives the estimated value of the total current travelling to the ground. The stronger the correspondence between the analytical and numerical values, the more the conditions of the simulation (as the morphology, electrical properties of the virtual model, and the computational methods) correspond in an analytical and in vivo perspective. The human 2 mm resolution, surface-based grounded model has been simulated under a uniform electric field of 1 kV/m 50 Hz. Our simulation with CST software gives 15.24 μA for Ansoft, and a 0.03% error, according to Equation (3).

## 3. Numerical Results

### 3.1. Induced Electric Field in Virtual Models without Implants

The induced electric fields in different tissues per 1 kV·m^−1^ of the 50 Hz external vertical electric field, are given in Table 1 for the mean values and Table 2 for the maximum values, following our simulations and other previous studies, in order to compare the results [19,20,32,33,34]. Uvic, Duke, and Taro are voxel models (hexaedral), whereas Maxwel and Ansoft are surface-based. The overall levels of amplitude in the literature are coherent. The key e differences in the results are comprised of morphological and dielectric parameter differences. Although different numerical methods are used in the literature, the influence of numerical methods can be neglected, according to [20].

The models Uvic [32], Duke [33], Taro [34] and Maxwel [19], are, respectively: 1m77, 1m74, 1m73, and 1m76, to be as close as possible to the reference male model [29]. Our model is 1m83 tall, representing a worse case concerning a pacemaker’s EMI. This slightly increases the currents going to ground, according to Equation (3), from approximately 8% to 12.5%, compared to the models cited above. However, one may not deduce a linear increase of inductions to the organs. The results also depend on the geometry of the organs. To underline the morphology’s crucial role: in the existing main literature, it is stated that blood inductions vary up to 26%, whereas skin inductions vary up to 49.7%, mainly because of vessel and skin surface representation divergence from one study to another [20].

Our model results present some particularity compared to the literature, for the morphological aspects. Our results for the higher organs, such as the brain, cerebellum, and bone (skull and shoulders), exhibit mean values that are greater than those in the literature, because of the height of our model. Moreover, Ansoft bone is more voluminous, due to the height. Our mean skin value is close to the value of the MAXWEL surfacique model and divergent to the DUKE Voxel model, and we presume that staircase effects amplify the values.

When considering the maximum values, in our surface-based model, some organs have a sharp-end when represented in tetrahedral, so the maximum value of the muscle in our study is greater than UVIC, which is fatter. Ansoft vessel representations do not reach the extremities of the body, such as the wrist and ankle, so the voxel representation of blood in those areas is superior to our values. Our spinal cord representation is more cylindrical and less detailed, when compared to TARO or Maxwell, which decrease the inductions. The brain in tetrahedral representation is smoother and circular, compared to UVIC and TARO hexahedral meshes, so our maximum values are lower.

To resume, the main aim of this study is to compute the EMI of pacemakers, whose influencing parameters are negligible for the heart region with a cardiac implant.

The inductions of 33 different organs for Ansoft are given in Table 3, for the mean and maximum values. Our results are coherent with the mean values in the literature. There are more differences in the maximal values and they are so sensitive to numerical errors that it is difficult to compare them. The staircase effects of hexahedral meshes on skin inductions add more variation to the results. The 99th percentile has a great utility for comparing the results of the voxel models. In our case, as we were using a surface-based model and tetrahedral meshing, we did not calculate the 99th percentile. The main aim of this study is to compute the induced voltage on pacemakers, whose divergences are negligible for the heart region with an implantation. We conclude that using the Ansoft 2 mm surface-based model is appropriate for this study.

### 3.2. Virtual Human Model with Pacemaker Implanted

More than three out of four worldwide pacemaker implantations are located in Europe and USA, where 99% of the lead implantations have right atrium (RA) or right ventricle (RV) bipolar configuration [4]. In addition to bipolar RA and RV, their unipolar configuration will also be considered in our work as a worst case configuration. The pacemaker model is designed in 3D, as a standard pacemaker box (4 cm × 3 cm × 0.5 cm), including a lead and connector part. It is implanted in the thorax of the Ansoft model in a particular manner, so that the lead is in contact with myocardia and the lead trajectory is fixed to 20 cm vertically and 8 cm horizontally. The dielectric property of the lead isolation is chosen as 10^−12^ S/m, with a permittivity of 6, and the lead electrodes, which are often made of platinum, are defined as 9.52 × 10^6^ S/m.

#### 3.2.1. Leads Dimensions

**Figure 2:**
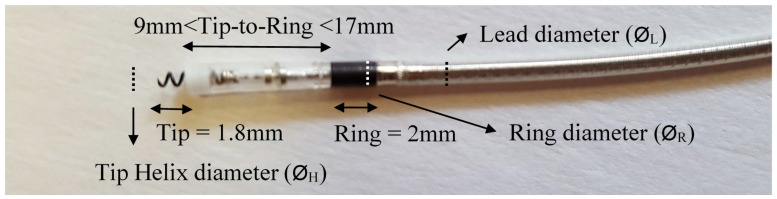
Active fixation pacemaker lead and its parts : Lead diameter (Ø_L_), Ring length (constant = 2 mm) and diameter (Ø_R_), tip to ring distance(9 mm to 17 mm), tip length (constant, 1.8 mm), and tip helix diameter (Ø_H_).

We found technical information on recent pacing leads on the websites of five diverse international manufacturers [35,36,37,38,39]. In this way, we aim to compare diverse representative cases on recent leads in the market. Multiple electrodes or multiple chamber configurations of pacemakers, are not considered. According to [40], 80% of recent leads are active fixation leads, which prevent some displacement problems, so passive fixation specific geometry will constitute part of the future studies. Table 4 presents the main models with their description, including two lead models for each of the five manufacturers. Figure 2 shows an illustration of the active fixation leads and their parts, to clarify/resume the dimensions mentioned in Table 4. As seen in Figure 2, most of the leads have a 2 mm ring length, their cable diameter is very similar to that of the ring electrode, and they have a 1.8 mm tip helix diameter, so these parameters will be valid for all simulations in the following sections. The lead diameter and ring diameter are very similar to each other, and there is less than a 0.1 mm difference between them. However, their dimensions vary from 4.1 Fr to 6.7 Fr (1.37 mm–2.23 mm), depending on the model. Independently, the helix diameter also varies, depending on the manufacturer or model. Another parameter is the surface of the helix and ring, expressed in mm^2^ in Table 4. This surface varies between models, in order to produce a more active surface to stimulate, by varying the surface shape, making it more porous. These parameters are very small to be able to simulate them numerically, and are protected by many patents. This study will not take the helix and ring surfaces into account.

#### 3.2.2. Induced Voltage Calculation

We simulated the human model implanted under 1 kV·m^−1^ of the 50 Hz exposition, always grounded by the feet under exactly the same conditions as previous parts. The induced voltage on the pacemaker leads is calculated by the integration on the induced electric field between the two electrodes.

**Figure 3:**
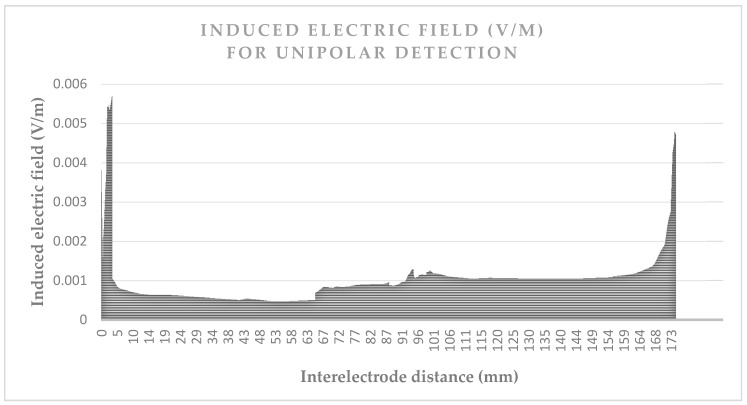
Induced electric field (V/m) between the electrodes (mm) in the unipolar detection of a pacemaker implanted to Ansoft 2 mm (grounded), due to the vertical electric field exposition (1 kV/m at 50 Hz).

**Figure 4:**
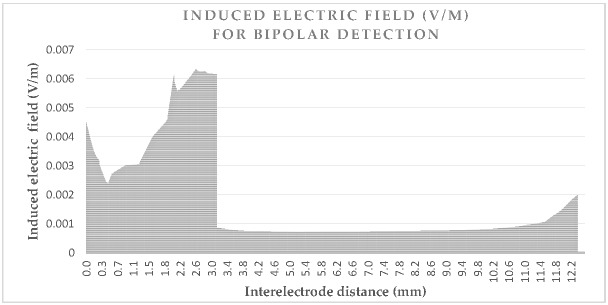
Induced electric field (V/m) between the electrodes (mm) for the bipolar detection of a pacemaker implanted to Ansoft 2 mm (grounded), due to the vertical electric field exposition (1 kV/m at 50 Hz).

Figure 3 and Figure 4 give the values of the induced electric field over the detection path, for a lead with a 10 mm tip-to-ring distance, a diameter of 6.7 Fr, and a tip-lead in a RV bottom position.

Figure 3 shows the induced electric field over the unipolar detection curve, which is about 180 mm. The values are approximately five times greater when near the box or tip, than the field values over the organs. Figure 4 shows the induced electric field bipolar detection curve, which also illustrates the same increase of five times, as may be observed for the tip; however, a smaller increase is observed for the ring which has the isolation nearby. The detection curve is just more than 12 mm and not exactly 10 mm, due to the necessity of a spine line between them, rather than a linear line. The influence of lead characteristics on the induced voltage will be hereafter studied, for different cases. We may already deduce from the curves that the detection curve is greater in a unipolar mode, than in a bipolar mode.

Following the same method, each different case of lead and implantation will be simulated, and the integral of their curves will illustrate the induced voltage, which will be discussed in the following sections.

#### 3.2.3. Influence of the Lead Dimensions

The length of the lead is kept constant for all simulations, in order to facilitate the comparison for different lead dimensions and tip-to-ring distances. The induced voltage comparison for the RV bottom position between the different diameters of lead cable (Ø_L_) and the helix diameters of the tip (Ø_H_), are shown in Table 5. We considered 24 different configurations; sensing mode: unipolar/bipolar, tip-to-ring distance: 9 mm/10 mm/17 mm, constant Ø_H_**:** minimum and maximum bipolar Ø_L_, maximum Ø_L_: 3 different Ø_H_).

The overall unipolar sensing mode results are almost uniform, as seen in Table 5. Tip-to-ring distance and cable dimension variations do not show any significant contrast for the unipolar detection. The maximum helix diameter increased the results by approximately 4 μV. Nevertheless, there is almost no change in the percentage (2%), which means that the increase of EMI is insignificant, compared to the other cases. This is coherent with the fact that the induced voltage is calculated in unipolar mode, for the distance between the box and the tip; a distance which is quite constant in these different configurations.

As expected, the tip-to-ring distance increases the induced voltage when in bipolar mode, by approximately 1 μV per each additional 1 mm. The influence of the helix/cable diameters is very slight, when compared to the tip-to-ring distance.

The unipolar interference voltages are 7.7 greater than the bipolar ones for RV when considered as a mean value, taking into account the 24 cases presented in Table 5.

#### 3.2.4. Influence of the Tip Position

**Figure 5:**
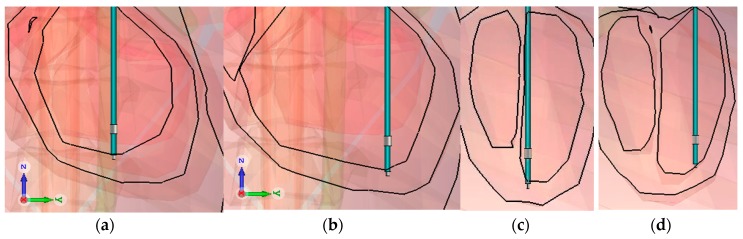
Implantation with the diverse position of the tip in the same chamber: (**a**) middle of the Right Ventricle in coronal view (**b**) bottom of the Right Ventricle in coronal view (**c**) middle of the Right Ventricle in sagittal view (**d**) bottom of the Right Ventricle in sagittal view.

The influence of the different position of the lead in RV was analyzed by considering two cases. Figure 4 shows the two positions: the coronal and sagittal view. First, only the bottom surface of the tip was in contact with the myocardia and was positioned as shown in Figure 5a,c, in the middle of the RV (Mid in Table 6). Secondly, all of the surface of the tip was included in the myocardia, as shown in Figure 5b,d at the bottom of the RV (Bottom in Table 6). The tip-to-ring distance is varied, in order to assess the impact.

The pacemaker box is transposed with the lead, so the lead length is kept the same. This facilitates the comparison by decreasing the morphological impact of the virtual model. Clinically, the box is fixed and the lead distance varies, depending on the pathology. So, the results of Table 6 should be treated with caution, and further studies are necessary to evaluate the morphological impacts and the real trajectory of the lead, by using diverse virtual human models.

For ventricular implantations, the distance between the T-R (bipolar) varies [36,37,38,39,40]. A 6.7 Fr lead cable diameter and 1.4 mm helix diameter are used to amplify the helix surfaces and the impacts. These two diverse positions and the T-R distance are investigated in Table 6.

For a pacemaker programmed in a unipolar sensing mode, with a constant lead length, and the movement of the box of the pacemaker, there is a 12% higher interference voltage in the RV bottom, than in the RV mid. For obvious anatomical reasons, the RV bottom is supposed to have a greater lead length than the RV mid, and that amplifies the percentage difference.

With the bipolar lead (containing two electrodes) and a pacemaker set to a unipolar sensing mode because of the clinical expectancy or a potential switch to a bipolar mode later on, the presence of the bipolar electrode may increase the interference voltage by 4 μV for the mid RV and 0.3 μV for the bottom RV. The tip-to-ring distance barely affects the unipolar sensing. There is a slight decrease (1–2 μV) in the unipolar-induced voltage as the tip-ring distance increases to 17 mm. Those voltages are negligible compared to the unipolar-induced voltages (158.8–178.4).

The bipolar sensing mode differs between the mid and bottom RV positions of the implantation: the induced voltages vary between 2.15 and 2.56 times. However, the bipolar detection mode is highly influenced by inductions inside the heart, and morphological factors are much more critical. For those reasons, the results have to be confirmed by another virtual human model, in order to clarify the impact of morphology.

#### 3.2.5. Chamber Impact on Induced Voltage

If one analyzes the induced electric field of the heart for a human without an implant, the blood, being highly conductive (0.7 S/m), makes a path for the current density, until it reaches the heart muscles (0.08 S/m). By following the vessels, currents are able to reach the inside the heart filled of blood, but face resistance at the frontier of the heart muscles. Some current densities follow the blood circulation once they have reached the heart muscle and a small amount of current density passes through it; in both cases making the current densities concentrate at the bottom of the heart, inside the chambers. Due to the volume of the ventricle, which is superior to the auricle, and the ventricle position, which is inferior to the auricle, the current densities in the chambers, and by ohm law, the induced electric fields, are more superior in the RV than in the RA. So, we may expect that the induced voltages over the tip implanted in the RV are superior to those in the RA.

**Figure 6:**
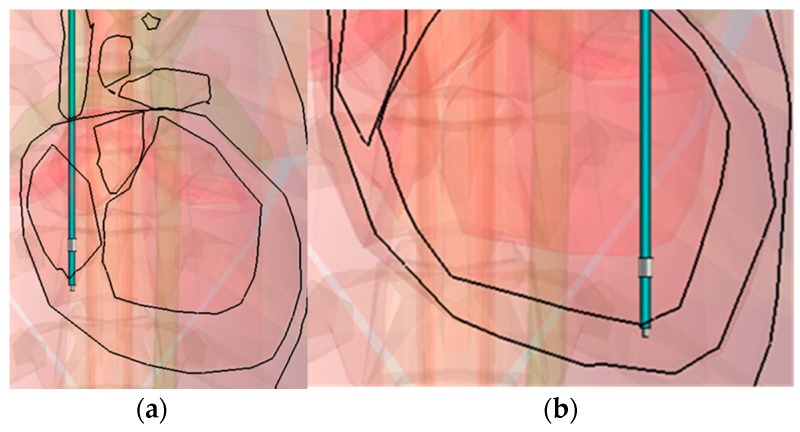
Tip implantation in the Right Atrium (**a**); and the Right Ventricle (**b**), both cases for the bottom implantation position of the chamber concerned in coronal view.

The tip-to-ring distance (T-R) is chosen to be 9 mm, to represent the usual active fixation configuration for atrium leads, which, according to the available data, is for a simple anatomical reason: the thickness of the cardiac muscle. A 4.1 Fr lead is chosen here to correspond with an atrium/ventricle designed model (3rd in Table 4). The results of the configuration of Figure 6 are presented in Table 7.

In both atrial and ventricular cases, we reveal a factor of almost 10, between unipolar and bipolar configuration. The factor is 12.8 for the RA and 8.7 for the RV, with a mean value of 10.7. Considering the same lead length, the induced voltage is 4% higher in RA unipolar configuration than in RV configuration. Plus, the pacemaker’s atrium maximum sensing voltages (0.2 mV) are set much lower than those of the ventricle (0.5 mV), which will increase their sensitivity to the 50 Hz electric field. However, these conclusions are only valid for unipolar configuration and announced sensitivity voltages which will be detailed in Table 8 and the following paragraph.

In bipolar configuration, the induced voltage is taken into account, and the RV bottom implantation has 1.41 times more induced voltages than RA implantation. In vitro studies point out that the sensitivity of atrial modes by using the same phantom for RV and RA, hasn’t been taken into consideration in this interference voltage variation. In order to solidify the result, different virtual human simulations have to be effectuated, to clarify that this percentage is not due to the morphology of Ansoft, and that it is valid for other anatomical models.

#### 3.2.6. Sensitivity Settings Influences

Induced voltage results will be a key-fact to estimate the interference threshold levels of the external electric field for the patient concerned, if the sensitivity voltage is known. For a bipolar sensing mode, if the maximal sensitivity (lowest voltage) of the pacemaker’s detection is considered as 0.2 mV, the threshold may be expected, according to the worst case scenario (27.6 μV), to be 7.24 kV·m^−1^. This level is ten times greater in a nominal sensitivity (2 mV) for pacemakers, being 72.4 kV·m^−1^, and we can estimate from that numerical result that they are immune to extremely high electric fields for that sensitivity.

If the implantable cardiovascular defibrillator (ICD) structure singularities (chock spire, box volume…) are neglected and we consider that the induce voltages are the same, for the RV bipolar mode’s maximum sensitivity (0.15 mV), the threshold may be estimated as 5.43 kV/m. The same estimation for the ICD RV bipolar mode’s nominal sensitivity (0.6 mV), will give a 21.72 kV·m^−1^ threshold of the external vertical electric field. However, these results have to be revaluated with simulations using an ICD 3D model.

Threshold estimations are given in Table 8, by taking into account the highest induced voltage value for each case, presented previously in Table 5, Table 6 and Table 7.

For an extremely rare case (<1%), previous unipolar detection mode’s maximal sensitivity, the voltage level varies from 0.5 mV to 1 mV for RV, and to 0.2 mV for RA. For a unipolar setup to a 1 mV maximal sensitivity, we can estimate a value of 5.6 kV·m^−1^ for the RV (178.4 μV). Using the same case for the RA, when the maximal sensitivity is taken as 0.2 mV, as it is for many pacemakers, we may estimate a value of 1.1 kV·m^−1^ for the RA (185.5 μV).

For the unipolar detection mode with a nominal sensitivity (2 mV), the threshold of dysfunction can be numerically estimated as 11.2 kV·m^−1^ for RV implantation (178.4 μV) and 10.8 kV·m^−1^ for RA implantation (185.6 μV).

If the sensitivity voltage is increasing, independently from the sensing mode, the thresholds of detection will increase linearly, in numerical estimations. As a result, the electric field thresholds for the bipolar mode’s nominal sensitivity often gives the possibility to implant workers, to regain their electric field exposed work places. However, to confirm that the induced voltage does not result in any dysfunction or noise in real pacemakers and defibrillators, comparative in vitro tests are needed.

## 4. Discussions

First, it should be acknowledged that these results are only valid for an electric field of 50 Hz. Our level of thresholds for a RV unipolar is calculated as 5.6 kV·m^−1^, and the value is in the same order as the dysfunction found in the in situ study on the pacemaker dysfunction level (6.7–7.5 kV/m) [41]. This experimental value is higher because pacemakers use electronic circuits and algorithms to detect and discriminate EMI from the biological heartbeat; such detail that we could not include in our simulations.

The same author has completed similar in situ ICD tests and detected disturbances to 5.1 kV·m^−1^, which are in agreement with our estimation of 5.6 kV·m^−1^ [42]. The value is higher in our numerical estimation because we supposed that ICD-induced voltages are similar to PM-induced voltages; however, supplementary simulations need to include choc spires and a more voluminous box.

Different lead parameters were compared to each other, and only tip-to-ring distances significantly changed the induced voltages for bipolar detection (Table 5). Using a unipolar (no ring electrode) or bipolar lead for unipolar detection did not influence the induced voltages (Table 6).

Another problem that our article aimed to treat was the trend of a smaller lead size, which is reconsidered, and even seen as clinically counterproductive, according to an Expert Review of Cardiovascular Therapy [43]. A numerical investigation of EMI depending on lead size is pursued for the RV implantation of 24 different cases. For unipolar and bipolar sensing modes, the influences of cable/helix diameters were insignificant when considering their EMI amplitude. Contrary to expectation, existing thin helix tips do have a lower influence on the induced voltages than a 1 mm additional tip-to-ring distance in the bipolar sensing mode.

Numerical analyses provide a good estimation of the subject, as shown in the first paragraph; however, those results need to be approved by complementary experiments, in order to observe the parameters that are not includable in the simulation due to computational limitations and intellectual property rights. The implantation position and the implantation chamber modify the value of the induced voltages, notably on the bipolar detection. The detection thresholds are also modified according the implantation of the chamber.

Our study aims to compare different configurations in cases which are well-defined and which can compared. The sensing voltages and other configurations of a pacemaker have to be taken into consideration to estimate the real EMI sensitivity of the patient. In vitro studies are necessary to analyse further parameter impacts on a pacemaker’s detections, as are in vivo studies for clinical aspects.

## 5. Conclusions

With the numerical FIT method, we investigated a surface-based human body model (2 mm resolution) to determine electric field inductions from an external 50 Hz electric field inside different organs. Based on the same validated numerical methods and human model, the possible interferences that may result due to those fields over active implantable medical devices, especially pacemakers, were numerically analysed.

As the main aim of study, the induced voltages caused by the external electric field were calculated on a pacemaker implanted human model. The different properties of the leads were studied and compared to each other, in order to demonstrate which parameters have a greater influence. There were no specific lead parameters influencing the induced voltages for unipolar detection. The same case was presented for bipolar detection, except for the tip-to-ring distance, which constitutes up to 46% of the influence (RV mid. implantation).

Different implantation positions (middle and bottom of RV) are also treated. The inductions diminish more considerably in the middle position than in the bottom position, for both detection modes. For unipolar detection, the decrease is max. 12%, contrary to bipolar detection, where the influences are so critical that they have to be verified with other anatomical models, in order to determine if the influences are due to the specific morphology of the virtual model used.

Different chamber implantations (RV and RA) are compared, according to the induced voltages. The RV has a 41% higher induced voltage than the RA, for the obvious reason of having a greater current density.

These results may help to identify possible thresholds dysfunction; estimations from our numerical investigations give a threshold of 7.24 kV/m for bipolar sensing mode, for a maximum sensitivity (0.2 mV). Covering 99% of implanted pacing leads in the USA and Europe, right atrium and right ventricle implantations are studied and compared to each other.

Considering the mean value of the induced voltage for the RA and RV, the unipolar sensing mode result is a 10.7 times greater induced voltage than the bipolar sensing mode.

If we consider the thresholds for nominal sensitivity for the RA and RV, the unipolar sensing mode results in a 9.55 times lesser threshold in kV/m, than the bipolar one. However, the factor is less important when comparing the maximal sensitivity, which varies between the detection chamber and the mode, making it difficult to compare the results. The trademark and model may define another maximal sensitivity voltage.

For perspective, these theoretical results are intended to form the basis of an experimental study to investigate the behaviour of different models with cardiac implants subjected to an electric field. Experimental in vitro tests and theoretical simulations will be compared to furnish an easy tool, as a complementary alternative to clinical studies.

## Figures and Tables

**Table 1:** Mean induced electric field (mV·m^−1^) per 1 kV·m^−1^ of the pure vertical electric field.

mV·m^−1^/kV·m^−1^	UVIC	MAXWEL	DUKE	ANSOFT
**Blood**	1.2	0.7		0.88
**Bone**		2.2		2.91
**Bone trabecular**		4.8		4.66
**Brain**	0.7			0.9
**Brain Grey Matter**		0.5	0.8	
**Brain White Matter**		0.8	0.9	
**Cerebellum**			1.1	1.59
**Heart**	1.0	1.0	1.1	1.14
**Marrow bone**	2.7			2.8
**Muscle**	1.4	1.3	1.4	1.25
**Skin**		1.9	3.8	1.43
**Spinal cord**		1.1	1.3	1.27

**Table 2:** Maximum induced electric field (mV·m^−1^) per 1 kV·m^−1^ of the pure vertical electric field.

mV·m^−1^/kV·m^−1^	UVIC	MAXWEL	TARO	ANSOFT
**Blood**	19.47			4.8
**Brain**	2.8		2.86	1.7
**Heart Lumen**	2.40			2.44
**Marrow bone**	30.4			30.6
**Muscle**	24.42			29.4
**Spinal cord**		6.74	7.81	4.5
**Eye humour**		0.333		0.26
**Optic nerve**		1.43		3.1

**Table 3:** Ansoft body model (2 mm resolution) top irradiation and grounded conditions, electric field induced on the organs (mV·m^−1^) due to 1 kV/m of the 50 Hz uniform electric field exposure [31].

mV·m^−1^/kV·m^−1^ Organ Name	Isotropic σ (S·m^−1^)	E_average_	E_max_
**Bile**	1.40	0.28	0.77
**Bladder**	3.3	0.8	1.41
**Blood**	0.70	0.88	4.8
**Bone cortical**	0.02	2.91	32.5
**Bone trabecular**	0.07	4.66	21.6
**Bone marrow**	0.10	1.95	30.6
**Brain**	0.08	0.90	1.7
**Cartilage**	0.17	0.98	2.38
**Cervellium**	0.08	1.59	2.45
**Eye (humour)**	1.50	0.15	0.26
**Eye (nerve)**	0.02	1.38	3.1
**Gall bladder**	0.9	1.36	3.2
**Heart muscle**	0.08	1.14	4
**Heart lumen**	0.70	0.545	2.44
**Intestine large**	1.20	0.673	2.9
**Intestine small**	1.09	0.614	1.96
**Kidney**	0.09	1.28	3.3
**Larynx**	0.52	2.45	2.8
**Liver**	0.07	1.78	3.95
**Lung (inflated)**	0.07	1.23	2.56
**Muscle**	0.35	1.25	29.4
**Oesophagus**	0.52	1.32	2.74
**Pancreas**	0.21	1.24	2.01
**Prostate**	0.42	1.34	3.35
**Skin**	0.1	1.43	77.3
**Spinal cord**	0.03	1.27	4.5
**Spleen**	0.09	1.75	3.01
**Stomach**	0.52	0.70	2.09
**Trachea**	0.3	1.36	2.19
**Trachea (inside)**	0	1.95	4.58
**Testis**	0.42	0.13	0.85
**Teeth**	0.02	2.64	4.3
**Tongue**	0.27	1.05	3.5

**Table 4:** 10 different active fixation lead models from five different trademarks, including two leads per manufacturer.

Active Fix.	Tip-Ring	Tip Helix	Ring	Diameter	Material
**Vitratron ICF 09B**	10 mm	1.8 mm 4.2 mm^2^	22 mm^2^	Ø_L_: 2.03 mm (6 Fr) Ø_R_: 1.98 mm Ø_H_: 1.17 mm	Platinized
**Vitratron ICQ 09B**	10 mm	1.8 mm 4.2 mm^2^	22 mm^2^	Ø_L_: 1.98 mm (5.7 Fr) Ø_R_: 1.98 mm Ø_H_: 1.17 mm	Titan. Ni. + Platinum
**Medtronic Selectsecure3830**	9 mm	1.8 mm		4.1Fr	Titan. Ni. + Platinum
**Medtronic CapSureFix Novus MRI SureScan**	10 mm	1.8 mm		6.1 Fr (model 5076)/5.7 Fr (model 4076)	Platinized/Titanium nitrate
**Boston Scientific Ingevity MRI**	-	-	-	6 Fr	-
**Boston Scientific Dextrus**	10 mm	-	-	6.7 Fr	-
**St Jude Tendril MRI LPA1200M**	10 mm	1.8 mm 6 mm^2^	16.5 mm^2^	6.6 Fr	Titanium + Platinum
**St Jude Tendril ST 1888/82 TC**	10 mm	2 mm 8.5 mm^2^	16 mm^2^	1.87 mm (1888) 2 mm Atrial (1882)	Titanium Ni. + Platinum
**Biotronic Setrox**	10 mm	1.8 mm 4.5 mm^2^	17.5 mm^2^	6.7 Fr Ring 6.6 Fr cable	Platinum/iridium
**Biotronic Solia**	10 mm	1.8 mm 4.5 mm^2^	17.4 mm^2^	5.6 Fr Ring 5.9 Fr Cable	Iridium

**Table 5:** Comparison between 24 different configurations; sensing mode: unipolar/bipolar, tip-to-ring distance: 9 mm/10 mm/17 mm, thin Ø_H_: minimum and maximum bipolar Ø_L_, maximum Ø_L_: 3 different Ø_H_) (per 1 kV·m^−1^ 50 Hz).

		Unipolar Interference Voltage (μV)				Bipolar Interference Voltage (μV)			
Diameters |T-R|:		9 mm	10 mm	17 mm	Mean	9 mm	10 mm	17 mm	Mean
**Ø_H_: 0.9 mm**	**Ø_L_: 4.1 Fr**	176.8	173.4	173.2	174.5	20.2	21.1	27.0	22.8
	**Ø_L_: 6.7 Fr**	174	174.3	175.7	174.7	20.7	21.34	27.6	23.2
**Ø_L_: 6.7 Fr**	**Ø_H_: 1.18 mm**	174.4	174.5	174.46	174.5	20.7	21.5	26	22.7
	**Ø_H_: 1.4 mm**	178.4	178.3	178.2	178.3	20.5	22.5	25.8	22.9

**Table 6:** Comparison between mid and bottom RV implantation concerning induced voltages (μV) produced on electrodes of pacemakers by the electric field (per 1 kV·m^−1^ 50 Hz).

		Unipolar induced Voltage (μV)				Bipolar induced Voltage (μV)			
|T-R|:	No Ring	9 mm	10 mm	17 mm	Mean	9 mm	10 mm	17 mm	Mean
**Mid**	158.8	161.8	162.8	160.7	161	8.2	8.83	12.0	9.7
**Bottom**	178.1	178.4	178.3	178.2	178.2	20.5	22.5	25.8	22.9

**Table 7:** Comparison between the right ventricle and atrium implantation, concerning the induced voltages (μV) on the electrodes of pacemakers during the 50 Hz electric field (per 1 kV·m^−1^).

	Unipolar Induced Voltage (μV)	Bipolar Induced Voltage (μV)
**Right Atrium**	185.6	14.5
**Right Ventricle**	178.4	20.5

**Table 8:** Electric field threshold (in kV/m, 50Hz) summary for different sensitivity and detection mode.

Detection Mode	Unipolar (kV/m)		Bipolar (kV/m)	
Sensitivity:	Maximal	Nominal (2 mV)	Maximal (0.2 mV)	Nominal (2 mV)
PM RV	5.6 (1 mV)	11.2	7.24	72.4
PM RA	1.08 (0.2 mV)	10.8	13.85	138
